# PDE 7 Inhibitors: New Potential Drugs for the Therapy of Spinal Cord Injury

**DOI:** 10.1371/journal.pone.0015937

**Published:** 2011-01-31

**Authors:** Irene Paterniti, Emanuela Mazzon, Carmen Gil, Daniela Impellizzeri, Valle Palomo, Myriam Redondo, Daniel I. Perez, Emanuela Esposito, Ana Martinez, Salvatore Cuzzocrea

**Affiliations:** 1 Department of Clinical and Experimental Medicine and Pharmacology, School of Medicine, University of Messina, Messina, Italy; 2 IRCCS Centro Neurolesi “Bonino-Pulejo”, Messina, Italy; 3 Instituto de Quimica Médica-CSIC, Madrid, Spain; National Institute on Aging Intramural Research Program, United States of America

## Abstract

**Background:**

Primary traumatic mechanical injury to the spinal cord (SCI) causes the death of a number of neurons that to date can neither be recovered nor regenerated. During the last years our group has been involved in the design, synthesis and evaluation of PDE7 inhibitors as new innovative drugs for several neurological disorders. Our working hypothesis is based on two different facts. Firstly, neuroinflammation is modulated by cAMP levels, thus the key role for phosphodiesterases (PDEs), which hydrolyze cAMP, is undoubtedly demonstrated. On the other hand, PDE7 is expressed simultaneously on leukocytes and on the brain, highlighting the potential crucial role of PDE7 as drug target for neuroinflammation.

**Methodology/Principal Findings:**

Here we present two chemically diverse families of PDE7 inhibitors, designed using computational techniques such as virtual screening and neuronal networks. We report their biological profile and their efficacy in an experimental SCI model induced by the application of vascular clips (force of 24 g) to the dura via a four-level T5–T8 laminectomy. We have selected two candidates, namely S14 and VP1.15, as PDE7 inhibitors. These compounds increase cAMP production both in macrophage and neuronal cell lines. Regarding drug-like properties, compounds were able to cross the blood brain barrier using parallel artificial membranes (PAMPA) methodology. SCI in mice resulted in severe trauma characterized by edema, neutrophil infiltration, and production of a range of inflammatory mediators, tissue damage, and apoptosis. Treatment of the mice with S14 and VP1.15, two PDE7 inhibitors, significantly reduced the degree of spinal cord inflammation, tissue injury (histological score), and TNF-α, IL-6, COX-2 and iNOS expression.

**Conclusions/Significance:**

All these data together led us to propose PDE7 inhibitors, and specifically S14 and VP1.15, as potential drug candidates to be further studied for the treatment of SCI.

## Introduction

Spinal cord injury (SCI) is a highly debilitating pathology [1]. Although innovative medical care has improved patient outcome, advances in pharmacotherapy for the purpose of decrease neuronal injury and promoting regeneration have been limited. The complex pathophysiology of SCI may explain the difficulty in finding a suitable therapy.

An excessive post-traumatic inflammatory reaction may play an important role in the secondary injury processes, which develop after SCI [2]. The primary traumatic mechanical injury to the spinal cord causes the death of a number of neurons that to date can neither be recovered nor regenerated. However, neurons continue to die for hours after SCI, and this represents a potentially avoidable event [3]. This secondary neuronal death is determined by a large number of cellular, molecular, and biochemical cascades. One such cascade that has been proposed to contribute significantly to the evolution of the secondary damage is the local inflammatory response in the injured spinal cord. Recent evidence, however, suggests that leukocytes, especially neutrophils which are the first leukocytes to arrive within the injured spinal cord [4], may also be directly involved in the pathogenesis and extension of spinal cord injury in rats. Several authors have demonstrated that neutrophils are especially prominent in a ‘marginal zone’ around the main area of injury and infarction at 24 h [5].

The cardinal features of inflammation, namely infiltration of inflammatory cells (not only polymorphonuclear neutrophils but also macrophage and lymphocytes), release of inflammatory mediators, and activation of endothelial cells leading to increased vascular permeability, edema formation, and tissue destruction have been widely characterized in animal models of SCI [6]. Both necrotic and apoptotic mechanisms of cell death after SCI then, have been well and extensively described in animal SCI models [7].

Phosphodiesterases (PDEs) are a large family of metallophosphohydrolase enzymes that ubiquitously metabolize the second messengers adenosine and guanosine 3′,5′-cyclic monophosphates (cAMP and cGMP) to their respective inactive 5′-monophosphates[8]. cAMP and cGMP are synthesized by adenylyl and guanylyl cyclases respectively, and mediate the action of hormones, neurotransmitters, and other cellular effectors in many physiologic processes. As elevation of intracellular cAMP level impacts immunosuppressive and anti-inflammatory properties [9], [10], selective inhibitors of cAMP-specific PDEs have been widely studied as therapeutics for the treatment of human diseases [11], predominantly immune disorders such as multiple sclerosis[12] and inflammatory processes [13], and also disorders of the central nervous system (CNS) such as depression, psychosis, and Alzheimer's disease[14].

**Figure 1:**
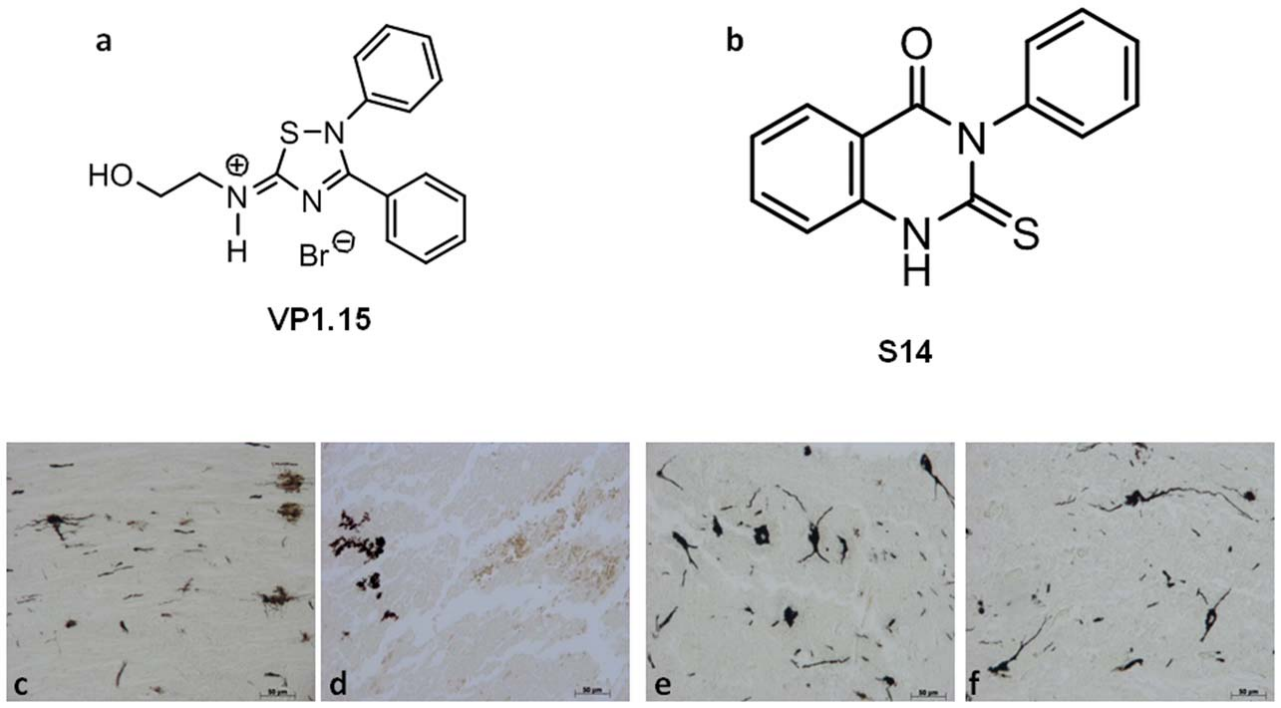
PDE7 inhibitors and their chemical structures. a and b represent a chemical structures of VP1,15 and S14respectively. In order in the spinal cord tissue collected from SCI mice, was observed the alteration of morphology of neurons when compared with sham-operated mice (Fig d and c respectively). A protection against alteration of neuron's morphology was observed in mice group treated with VP 1.15 and S14 (e, f respectively).

To date, most of the research has been centered on PDE4 inhibitors because PDE4 represents the major isoenzyme in most T-cell preparations and its selective inhibitors are able to decrease inflammatory cytokine production [15], [16]. PDE4 inhibitors have been widely studied as anti-inflammatory agents for the treatment of inflammatory disease and multiple sclerosis [17]. However, a major drawback of these compounds is the significant side effects such as emesis. To overcome these adverse effects, several strategies to dissociate the beneficial and detrimental effects of PDE4 inhibitors have led to some degree of success and the second generation of PDE4 inhibitors have shown better pharmacokinetic profiles[18]. An alternative approach is to target other cAMP-specific PDE families that are expressed in pro-inflammatory and immune cells. Initial evidence indicated that PDE7 had an important role in the activation of T-cells [19], [20]. However, results based on the use of PDE7A knockout mice (PDE7A^_^/^_^) failed to confirm the role of PDE7A in T-cell proliferation and suggested that this phosphodiesterase could have some other role in the regulation of humoral immune responses [21]. Thus, selective PDE7A inhibitors would be essential to elucidate the true potential of PDE7A as a pharmacological target in the context of the immune and neurological responses [22], [23]. The latest scientific findings concerning PDE7 and PDE4 inhibition suggest that selective small-molecule inhibitors of both enzymes could provide a novel approach to treat a variety of immunological diseases. In this context, our ligand-based virtual screening studies allowed us to identify quinazoline derivatives as a new class of PDE7 inhibitors [24]. This new family of inhibitors increases cAMP production both in macrophage and neuronal cell lines and reduces the inflammatory response induced by lipopolysacharide treatment in both types of cells cultures [25]. More recently, we have developed a neuronal network able to predict PDE7 inhibition activity of new molecules [26]. Using this drug discovery computational model, we have shown the PDE7 inhibitory properties of the 5-imino-1,2,4-thiadiazole heterocyclic family [27]. Here we present pharmacological properties of two chemically diverse families of PDE7 inhibitors (see chemical structure in Fig. 1a and b), designed using computational techniques such as virtual screening and neuronal networks. We report their CNS penetration properties, and their efficacy in an experimental SCI model. In particular, we have determined the following endpoints of the inflammatory response: (1) histological damage, (2) motor recovery, (3) neutrophil infiltration, (4) NF-κB expression, (5) iNOS formation, (6) pro-inflammatory cytokines production, and (7) apoptosis as Bax and Bcl-2 expression.

## Materials and Methods

### Animals

Male adult CD1 mice (25–30 g, Harlan Nossan, Milan, Italy) were used for all studies. Mice were housed in individual cages (5 for each group) and maintained under 12∶12 light-dark cycle at 21±1°C and 50±5% humidity. The animals were acclimated to their environment for 1 wk and had *ad libitum* access to tap water and standard rodent standard diet. All animal experiments complied with regulations in Italy (D.M. 116192), Europe (O.J. of E.C. L 358/1 12/18/1986) and USA (Animal Welfare Assurance No A5594-01, Department of Health and Human Services, USA). All behavioral testing was conducted in compliance with the NHI laboratory animal care guidelines and with protocols approved by the Institutional Animal Care and Use Committee (Council directive # 87-848, October 19, 1987, Ministère de l'Agriculture et de la Forêt, Service Vétérinaire de la Santé et de la Protection Animale, permission # 92-256 to SC). The study was approved by the University of Messina Review Board for the care of animals (PRIN ID 1042).

### SCI

Mice were anaesthetized using chloral hydrate (400 mg/kg body weight). We used the clip compression model described by Rivlin and Tator [28] and produced SCI by extradural compression of a section of the SC exposed via a four-level T5-T8 laminectomy, in which the prominent spinous process of T-5 was used as a surgical guide. A six-level laminectomy was chosen to expedite timely harvest and to obtain enough SC tissue for biochemical examination. With the aneurysm clip applicator oriented in the bilateral direction, an aneurysm clip with a closing force of 24 g was applied extradurally at T5-T-8 level. The clip was then rapidly released with the clip applicator, which caused SC compression. In the injured groups, the cord was compressed for 1min. Following surgery, 1.0 cc of saline was administered subcutaneously in order to replace the blood volume lost during the surgery. During recovery from anesthesia, the mice were placed on a warm heating pad and covered with a warm towel. The mice were singly housed in a temperature-controlled room at 27°C for a survival period of 10 days. Food and water were provided to the mice ad libitum. During this time period, the animals' bladders were manually voided twice a day until the mice were able to regain normal bladder function. Sham injured animals were only subjected to laminectomy.

### Experimental Design

Mice were randomized into 6 groups (N = 40 animals/group).

Sham animals were subjected to the surgical procedure except that the aneurysm clip was not applied and treated intraperitoneally (i.p.) with vehicle (10% DMSO) or S14 (10 mg/kg) or VP1.15 (4 mg/kg) 1 h, 3 h and 6 h after surgical procedure. The remaining mice were subjected to SCI (as described above) and treated with an i.p. bolus of vehicle (10% DMSO) or S14 (10 mg/kg) or VP1.15 (4 mg/kg) 1 h, 3 h and 6 h after SCI. The doses of S14 or VP1.15 used here were based on their PDE7 IC50 values (5.5 and 1.1 µM, respectively) and our experience on previous studies [29]. To investigate the motor score, in other set of experiments, the animals were treated with S14 (10 mg/kg) or VP1.15 (4 mg/kg) 1 h, 3 h and 6 h after SCI and daily until day 9. Ten mice from each group were sacrificed at different time points in order to collect samples for the evaluation of the parameters as described below.

### Myeloperoxidase activity

Myeloperoxidase (MPO) activity, an indicator of polymorphonuclear leukocyte (PMN) accumulation, was determined in the spinal cord tissues as previously described [30] at 24 hours after SCI. At the specified time following SCI, spinal cord tissues were obtained and weighed and each piece homogenized in a solution containing 0.5% (w/v) hexadecyltrimethyl-ammonium bromide dissolved in 10 mM potassium phosphate buffer (pH 7) and centrifuged for 30 min at 20,000 x g at 4°C. An aliquot of the supernatant was then allowed to react with a solution of 1.6 mM tetramethylbenzidine and 0.1 mM H_2_O_2_. The rate of change in absorbance was measured spectrophotometrically at 650 nm. MPO activity was defined as the quantity of enzyme degrading 1 µmol of peroxide per min at 37°C and was expressed as units of MPO/mg of proteins.

### Immunohistochemical localization of IL-1β, TNF-α, iNOS and COX-2

At the end of the experiment, the tissues were fixed in 10% (w/v) PBS-buffered formaldehyde and 8 µm sections were prepared from paraffin embedded tissues. After deparaffinization, endogenous peroxidase was quenched with 0.3% (v/v) hydrogen peroxide in 60% (v/v) methanol for 30 min. The sections were permeabilized with 0.1% (w/v) Triton X-100 in PBS for 20 min. Non-specific adsorption was minimized by incubating the section in 2% (v/v) normal goat serum in PBS for 20 min. Endogenous biotin or avidin binding sites were blocked by sequential incubation for 15 min with biotin and avidin, respectively. Sections were incubated overnight with anti-COX-2 antibody (Santa Cruz Biotechnology, 1∶500 in PBS, v/v), anti-TNF-α ligand antibody (Santa Cruz Biotechnology, 1∶500 in PBS, v/v), anti-IL-1β ligand antibody (Santa Cruz Biotechnology, 1∶500 in PBS, v/v), anti-iNOS antibody (Santa Cruz Biotechnology, 1∶500 in PBS, v/v). Sections were washed with PBS, and incubated with secondary antibody. Specific labeling was detected with a biotin-conjugated goat anti-rabbit IgG and avidin-biotin peroxidase complex (Vector Laboratories, DBA).

In order to verify the binding specificity for IL-1β, TNF-α, iNOS and COX-2 some sections were also incubated with only the primary antibody (no secondary) or with only the secondary antibody (no primary). In these situations no positive staining was found in the sections indicating that the immunoreaction was positive in all the experiments carried out. Immunocytochemistry photographs (n = 5) were assessed by densitometry. The assay was carried out by using Optilab Graftek software on a Macintosh personal computer (CPU G3-266). All the immunocytochemistry analysis was carried out without knowledge of the treatments.

### Western blot analysis for IκB-α, Bax, Bcl-2 and pERK-1/2

Cytosolic and nuclear extracts were prepared as previously described [31] with slight modifications. Briefly, spinal cord tissues from each mouse were suspended in extraction Buffer A containing 0.2 mM PMSF, 0,15 µM pepstatin A, 20 µM leupeptin, 1 mM sodium orthovanadate, homogenized at the highest setting for 2 min, and centrifuged at 1,000 x g for 10 min at 4°C. Supernatants represented the cytosolic fraction. The pellets, containing enriched nuclei, were re-suspended in Buffer B containing 1% Triton X-100, 150 mM NaCl, 10 mM TRIS-HCl pH 7.4, 1 mM EGTA, 1 mM EDTA, 0,2 mM PMSF, 20 µm leupeptin, 0,2 mM sodium orthovanadate. After centrifugation 30 min at 15,000 x g at 4°C, the supernatants containing the nuclear protein were stored at −80 for further analysis. The levels of IκB-α, Bax, and Bcl-2 were quantified in cytosolic fraction from spinal cord tissue collected after 24 hours after SCI. The filters were blocked with 1x PBS, 5% (w/v) non fat dried milk (PM) for 40 min at room temperature and subsequently probed with specific Abs IκB-α (Santa Cruz Biotechnology, 1∶1000), or anti-Bax (1∶500; Santa Cruz Biotechnology), or anti-Bcl-2 (1∶500; Santa Cruz Biotechnology), in 1x PBS, 5% w/v non fat dried milk, 0.1% Tween-20 (PMT) at 4°C, overnight. Membranes were incubated with peroxidase-conjugated bovine anti-mouse IgG secondary antibody or peroxidase-conjugated goat anti-rabbit IgG (1∶2000, Jackson ImmunoResearch, West Grove, PA) for 1 h at room temperature. To ascertain that blots were loaded with equal amounts of proteic lysates, they were also incubated in the presence of the antibody against β-actin protein (1∶10,000 Sigma-Aldrich Corp.). The relative expression of the protein bands of IκB-α (∼37 kDa), Bax (∼23 kDa), Bcl-2 (∼29 kDa) was quantified by densitometric scanning of the X-ray films with GS-700 Imaging Densitometer (GS-700, Bio-Rad Laboratories, Milan, Italy) and a computer program (Molecular Analyst, IBM), and standardized for densitometric analysis to β-actin levels. The dual-phosphorylated form of ERK (p-ERK) antibody identified two bands of approximately 44 and 42 kDa (corresponding to p-ERK1 and p-ERK2, respectively). The anti-ERK2 antibody detects total ERK2 (*i.e.* detects both phosphorylated and nonphosphorylated forms of ERK2).

### Light microscopy

Spinal cord tissues were taken at 24 h following trauma. Tissue segments containing the lesion (1 cm on each side of the lesion) were paraffin embedded and cut into 5-µm-thick sections. Tissue sections (thickness 5 µm) were deparaffinized with xylene, stained with Haematoxylin/Eosin (H&E), with methyl green pyronin staining (used to simultaneously DNA and RNA) and studied using light microscopy (Dialux 22 Leitz).

The segments of each spinal cord were evaluated by an experienced histopathologist. Damaged neurons were counted and the histopathologic changes of the gray matter were scored on a 6-point scale [32]: 0, no lesion observed, 1, gray matter contained 1 to 5 eosinophilic neurons; 2, gray matter contained 5 to 10 eosinophilic neurons; 3, gray matter contained more than 10 eosinophilic neurons; 4, small infarction (less than one third of the gray matter area); 5, moderate infarction; (one third to one half of the gray matter area); 6, large infarction (more than half of the gray matter area). The scores from all the sections from each spinal cord were averaged to give a final score for individual mice. All the histological studies were performed in a blinded fashion.

### Golgi impregnation

FD Neurotech kit (FD NeuroTechnologies, Ellicott City, Md, USA) was used for Golgi impregnation of spinal cord tissue. Blocks were placed directly into solutions A and B, without rinsing, and remained there for 2 weeks in the dark at room temperature. Forty-eight hours after placing the blocks in solution C (4°C), the blocks were frozen on dry ice and stored at −70°C until sectioning. Cryostat sections (100 µm) were cut at −25°C and mounted onto gelatinized slides. Slides were allowed to dry in the dark, and the rest of the staining process done as previously described.

Neurons chosen for tracing met the following criteria: (1) they were completely impregnated with Golgi stain, (2) they were unobscured by other impregnated neurons or precipitant, (3) 70% of the dendritic tree was visible within the plane of focus, and (4) dentate granule neurons must have been located in the outer one-half of the granule cell layer in DG. Cells chosen for analyses had to be well impregnated, clearly distinguishable from adjacent cells and have continuous unbroken dendrites. Spines were counted under oil (X100), using light microscopy (Axostar Plus equipped with Axio-Cam MRc, Zeiss), and the entire visible dendritic length measured by Imaging computer program (Axio-Vision, Zeiss). Spine density was calculated referring to the length of the dendrite.

### Measurement of spinal cord TNF-α and IL-1β levels

Portions of spinal cord tissues, collected at 24 hours after SCI, were homogenized as previously described in PBS containing 2 mmol/L of phenyl-methyl sulfonyl fluoride (PMSF, Sigma Chemical Co.) and tissue TNF-α and IL-1β levels were evaluated. The assay was carried out by using a colorimetric, commercial kit (Calbiochem-Novabiochem Corporation, USA) according to the manufacturer instructions. All TNF-α and IL-1β determinations were performed in duplicate serial dilutions.

### Grading of motor disturbance

The motor function of mice subjected to compression trauma was assessed once a day for 20 days after injury. Recovery from motor disturbance was graded using the Basso Mouse Scale [33].

### CNS drug penetration: *In vitro* Parallel artificial membrane permeability assay (PAMPA)

Prediction of the brain penetration was evaluated using a parallel artificial membrane permeability assay (PAMPA) [34]. Ten commercial drugs, phosphate buffer saline solution at pH 7.4 (PBS), DMSO and dodecane were purchased from Sigma, Acros organics, Aldrich and Fluka. The porcine polar brain lipid (PBL) (catalog no. 141101) was from Avanti Polar Lipids. The donor plate was a 96-well filtrate plate (Multiscreen® IP Sterile Plate PDVF membrane, pore size is 0.45 µM, catalog no. MAIPS4510) and the acceptor plate was an indented 96-well plate (Multiscreen®, catalog no. MAMCS9610 from Millipore). Filter PDVF membrane units (diameter 30 mm, pore size 0.45 µm) from Symta were used to filtered the samples. A 96-well plate UV reader (Thermoscientific, Multiskan spectrum) was used for the UV measurements. Test compounds (3–5 mg of Cafeine, Enoxacine, Hydrocortisone, Desipramine, Ofloxacine, Piroxicam, Testosterone, 12 mg of Promazine and 25 mg of Verapamile and Atenolol) were dissolved in DMSO (250 µL). 25 microlitres of this compound stock solution was taken and 225 µL of DMSO and 4750 µL of PBS pH = 7.4 buffer were added to reach 5% of DMSO concentration in the experiment. This solution was filtered. The acceptor 96-well microplate was filled with 180 µL of PBS/DMSO (95/5). The donor 96-well plate was coated with 4 µL of porcine brain lipid in dodecane (20 mg mL^−1^) and after 5 minutes, 180 µL of each compound solution was added. 1–2 mg of every compound to be determined their ability to pass the brain barrier were dissolved in 250 µL of DMSO and 4750 µL of PBS pH = 7.4 buffer, filtered and then added to the donor 96-well plate. Then the donor plate was carefully put on the acceptor plate to form a “sandwich”, which was left undisturbed for 4 h at 25°C. During this time the compounds diffused from the donor plate through the brain lipid membrane into the acceptor plate. After incubation, the donor plate was removed. The concentration of compounds and commercial drugs in the acceptor and the donor wells was determined by UV plate reader. Every sample was analyzed at three to five wavelengths, in 3 wells and in three independent runs. Results are given as the mean [standard deviation (SD)] and the average of the three runs is reported. 10 quality control compounds (previously mentioned) of known BBB permeability were included in each experiment to validate the analysis set.

### Materials

All compounds were obtained from Sigma-Aldrich Company Ltd. (Milan, Italy). All other chemicals were of the highest commercial grade available. All stock solutions were prepared in non-pyrogenic saline (0.9% NaCl; Baxter, Italy, UK). S14 and VP1.15 were synthesized in the Instituto de Quimica Medica following described procedures.

### Statistical evaluation

All values in the figures and text are expressed as mean ± standard error of the mean (SEM) of N observations. For the in vivo studies N represents the number of animals studied. In the experiments involving histology or immunohistochemistry, the figures shown are representative of at least three experiments performed on different experimental days. The results were analyzed by one-way ANOVA followed by a Bonferroni post-hoc test for multiple comparisons. A p-value of less than 0.05 was considered significant. BBB scale data were analyzed by the Mann-Whitney test and considered significant when p- value was <0.05.

## Results

### S14 and VP 1.15 reduce the severity of spinal cord trauma

**Figure 2:**
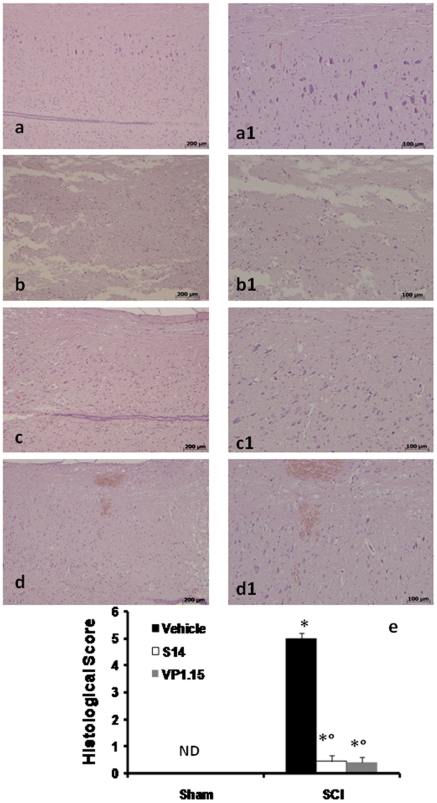
Effect of PDE7 inhibitors treatment on histological alterations of the spinal cord tissue 24 h after injury. A significant damage to the spinal cord, from SCI operated mice at the perilesional area, was assessed by the presence of edema as well as alteration of the white matter 24 h after injury (b and b1, see histological score e). Notably, a significant protection from SCI damage was observed in the tissue collected from VP1.15 and S14 treated mice (c, c1 and d, d1 respectively, see histological score e). The histological score (**e**) was made by an independent observer. wm: White matter; gm: gray matter. This figure is representative of at least 3 experiments performed on different experimental days. Values shown are mean ± s.e. mean of 10 mice for each group. *p<0.01 vs. Sham. °*p*<0.01 vs. SCI+vehicle.

The severity of the trauma at the level of the perilesional area, that assessed the presence of edema as well as alteration of the white matter, was evaluated at 24 h after injury. A significant damage to the spinal cord was observed in the spinal cord tissue from SCI mice when compared with sham-operated mice (Figures 2b,b1 and 2a,a1 respectively, see histological score Figure 2e). Notably, a significant protection against the spinal cord injury was observed in mice group treated with VP1.15 and S14 (Figures 2c,c1 and 2d,d1 respectively, see histological score Figure 2e). Alteration of the morphology of neurons and glia cells were observed by Golgi staining that selectively impregnates single neurons with silver chromate. A significant alteration of morphology was observed in the spinal cord tissue from SCI mice when compared with sham-operated mice (Figures 1c and 1d respectively). A protection against alteration of neuron's morphology was observed in mice group treated with VP1.15 and S14 (Figures 1e, and 1f respectively).

**Figure 3:**
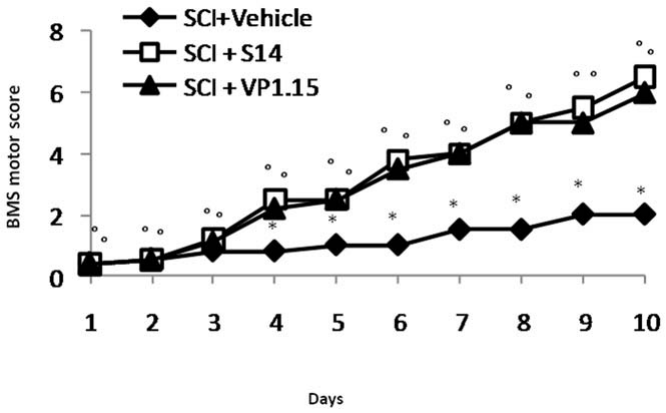
Effect of VP1.15 and S14 treatment on hind limb motor disturbance after spinal cord injury. The degree of motor disturbance was assessed every day until 10 days after SCI, by using the Basso mouse scale (BMS) open-field score. Treatment with VP1.15 and S14 reduces the motor disturbance after SCI. Values shown are mean ± s.e. mean of 10 mice for each group. *p<0.01 vs. Sham. °*p*<0.01 vs. SCI+vehicle.

Moreover to evaluate if histological damage to the spinal cord was associated with a loss of motor function, the modified BMS hind limb locomotor rating scale score was evaluated. While motor function was only slightly impaired in sham mice, mice subjected to SCI had significant deficits in hind limb movement (Figure 3). VP1.15 and S14 treatment ameliorated the functional deficits induced by SCI (Figure 3).

### Effect of VP 1.15 and S14 on IκB-α degradation

By western blot analysis we evaluated IκB-α degradation, to investigate the cellular mechanisms by which treatment with VP1.15 and S14 may attenuate the development of SCI.

**Figure 4:**
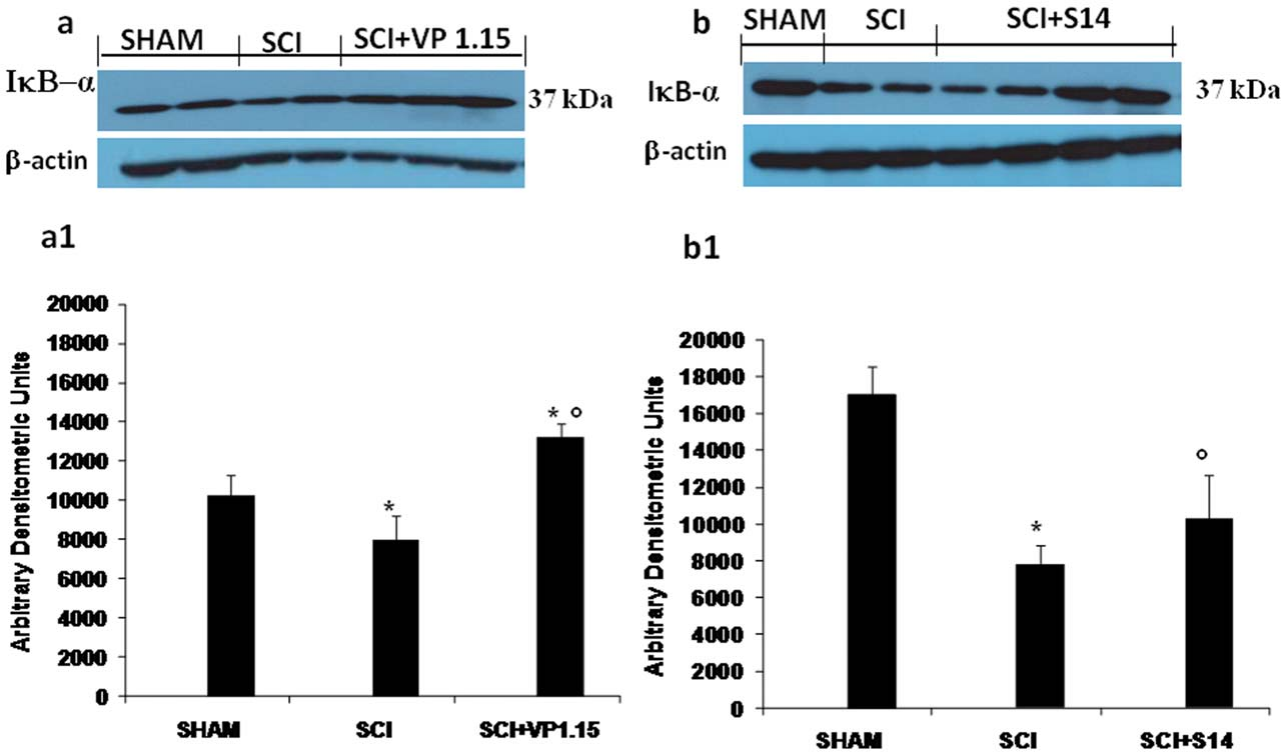
Effects of VP1.15 and S14 treatment on IκB-α degradation. By Western Blot analysis, a basal level of IκB-α was detected in the spinal cord from sham-operated animals, whereas IκB-α levels were substantially reduced in SCI mice (a, a1 and b,b1). VP 1.15 and S14 treatment prevented the SCI-induced IκB-α degradation (a, a1 and b,b1 respectively). A representative blot of lysates obtained from each group is shown, and densitometry analysis of all animals is reported. The relative expression of the protein bands from three separated experiments was standardized for densitometry analysis *P<0.01 *vs.* Sham; °*P*<0.01 *vs.* SCI.

A basal level of IκB-α was detected in the spinal cord from sham-operated animals (Figures 4a, a1 and 4b, b1), whereas IκB-α levels were substantially reduced in SCI mice. Administration of VP1.15 (Figure 4a, a1) and S14 (Figure 4b, b1) prevented the SCI-induced IκB-α degradation.

### Effect of VP 1.15 and S14 on expression of TNF-α and IL-1β and neutrophil infiltration after SCI

**Figure 5:**
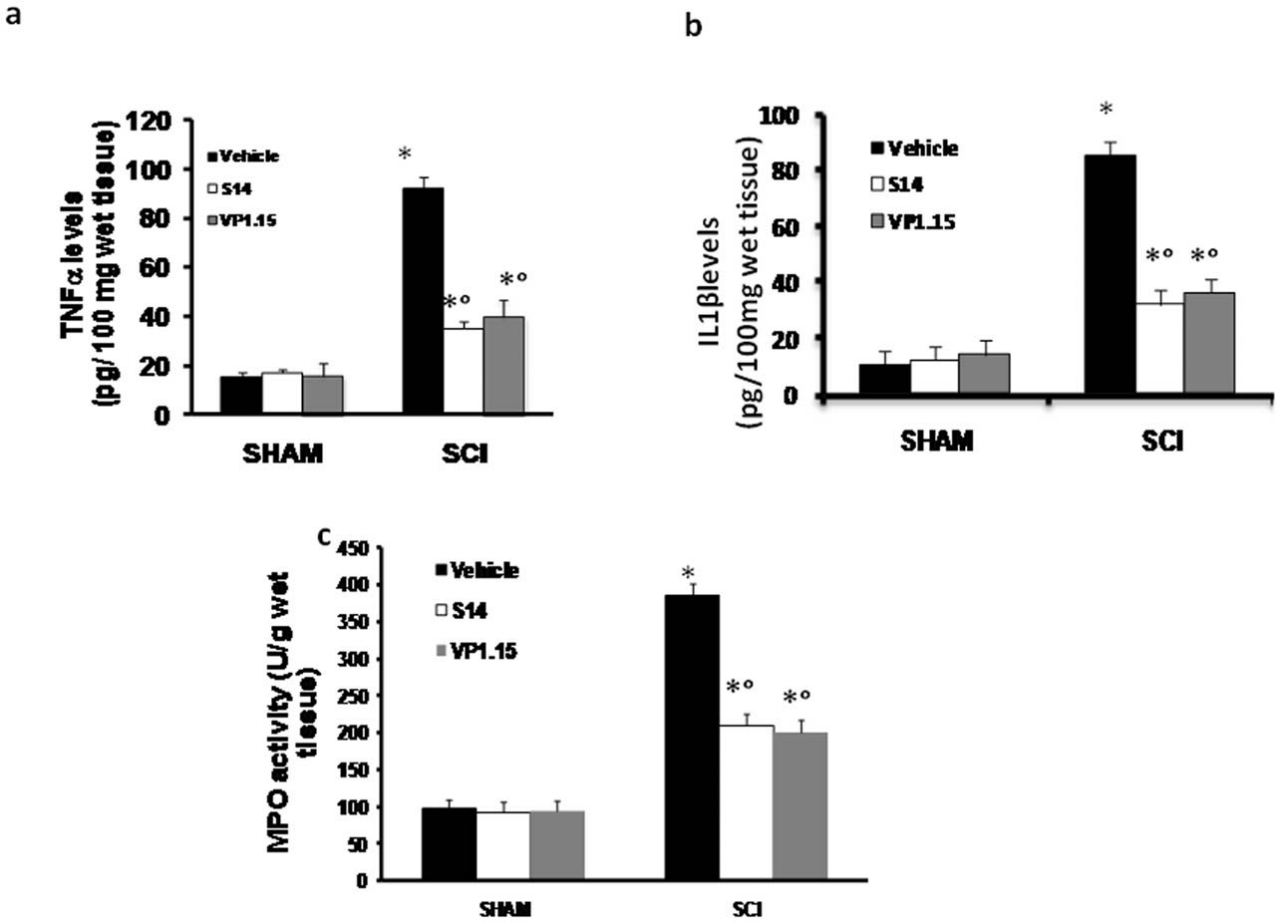
Effect of VP1.15 and S14 on TNF-α and IL-1β release and on MPO activity. A substantial increase in TNF-α (a) and IL-1β (b) production was found in spinal cord tissue collected from SCI mice at 24 h. Spinal cord levels of TNF-α and IL-1β were significantly attenuated by the VP 1.15 and S14 treatment (a, b respectively). Moreover, MPO activity in spinal cord of untreated SCI-operated mice was significantly increased at 24 h after the damage in comparison to sham mice (c). Treatment with VP 1.15 and S14 significantly reduced the SCI-induced increase in MPO activity (c). Data are means ±S.E. mean of 10 mice for each group. *p<0.01 vs sham, °*p*<. 0.01 vs SCI.

The histological pattern of spinal cord injury appeared to be correlated with the influx of leukocytes into the spinal cord. Therefore, we investigated the effect of VP1.15 and S14 on the neutrophil infiltration by measuring tissue MPO activity (Figure 5c). MPO activity was significantly elevated in the spinal cord at 24 h after injury in mice subjected to SCI when compared with Sham-operated mice (Figure 5c). In VP1.15 and S14 treated mice, the MPO activity in the spinal cord at 24 h after injury was significantly attenuated in comparison to that observed in SCI controls (Figure 5c).

To test whether PDE7 inhibitors modulates the inflammatory process trough the regulation of secretion of pro-inflammatory cytokines, we analysed the spinal cord levels of TNF-α and IL-1β. A substantial increase in TNF-α (Figure 5a) and IL-1β (Figure 5b) production was found in spinal cord tissue collected from SCI mice at 24 h. Spinal cord levels of TNF-α and IL-1β were significantly attenuated by the VP1.15 and S14 treatment (Figure 5a and 5b respectively).

### PDE7 inhibitors modulate the expression of TNF-α and IL-1β after SCI

**Figure 6:**
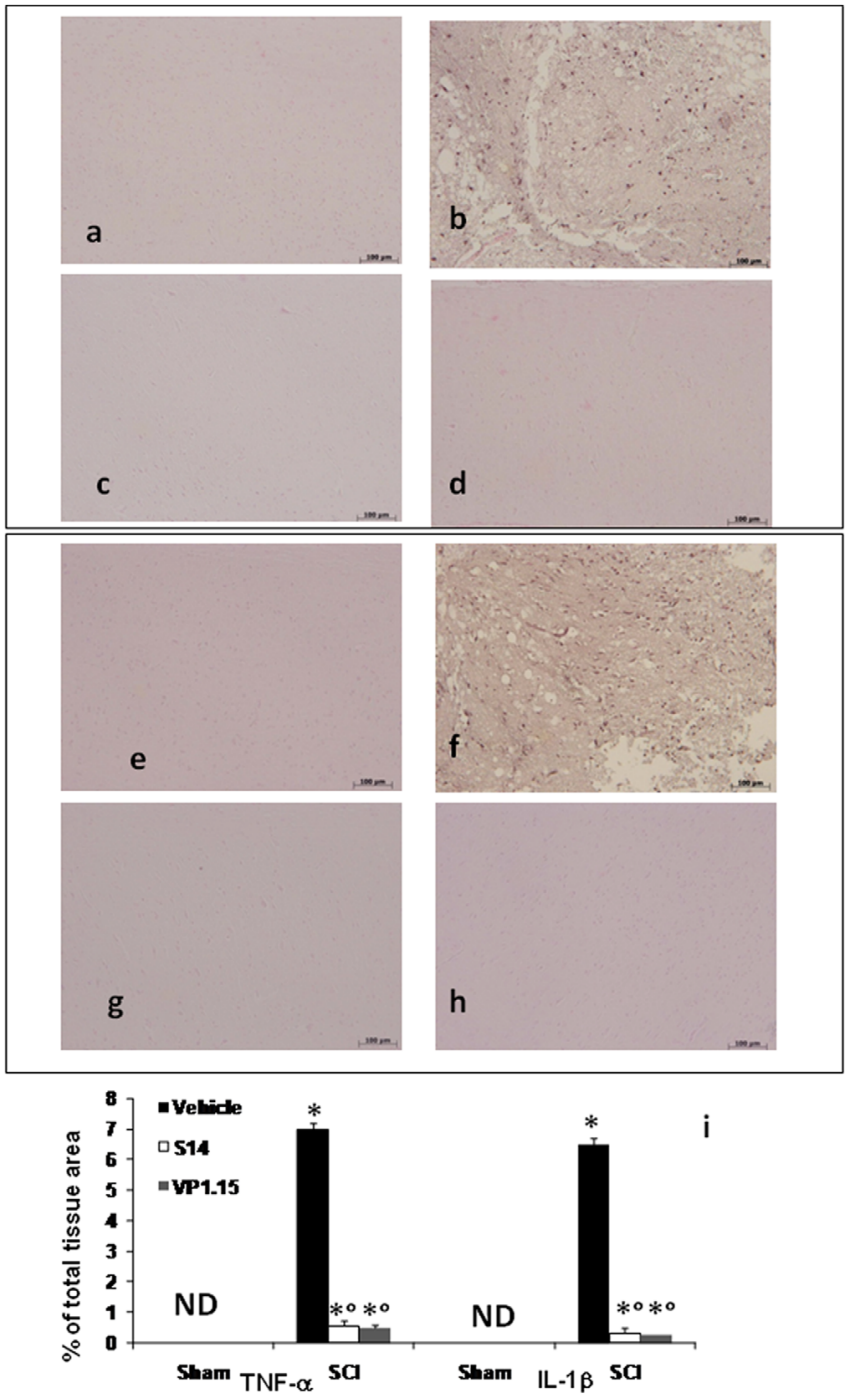
Effects of VP1.15 and S14 on TNF-α and IL-1β expression. In addition, spinal cord sections were processed at 24 h after SCI to determine the immunohistological staining for TNF-α and IL-1β expression. A substantial increase in TNF-α (b) and IL-1β (f) expression was found in inflammatory cells of the spinal cord tissues from SCI mice at 24^th^ hour after SCI. Spinal cord levels of TNF-α and IL-1β expression were significantly attenuated in VP 1.15 (d,h respectively) and S14 (c, g respectively) treated mice in comparison to SCI animals. Densitometry analysis of immunocytochemistry photographs (n = 5 photos from each sample collected from all mice in each experimental group) for TNF-α and IL-1β (**i**) from spinal cord tissues was assessed. The assay was carried out by using Optilab Graftek software on a Macintosh personal computer (CPU G3-266). Data are expressed as % of total tissue area. This figure is representative of at least 3 experiments performed on different experimental days. *p<0.01 vs. Sham. °*p*<0.01 vs SCI+vehicle. ND: not detectable

Moreover, spinal cord sections were taken at 24 h after SCI to determine the immunohistological staining for TNF-α and IL-1β expression. There was no staining for TNF-α Figure 6a) and IL-1β (Figure 6e) in spinal cord obtained from the sham mice. A substantial increase in TNF-α (Figure 6b, see densitometry analysis Figure 6i) and IL-1β (Figure 6f, see densitometry analysis Figure 6i) expression was found in inflammatory cells in the white and gray matter of the spinal cord tissues collected from SCI mice 24 hours after SCI. Spinal cord expression of TNF-α and IL-1β were significantly attenuated in mice treated with VP1.15 (Figure 6d and 6h respectively, see densitometry analysis Figure 6i) and S14 (Figure 6c and 6g respectively, see densitometry analysis Figure 6i) in comparison to SCI animals.

### Effect of VP1.15 and S14 on COX-2 expression and on activation of MAPK signal transduction pathway

As phosphorylation of ERK1/2 results in expression of genes, such as that encoding for COX-2, mediating the inflammatory responses characteristic of SCI. The expression of this enzyme and ERK1/2 phosphorylation in homogenates of spinal cord tissues was investigated by western blot and immunohistochemical analysis at 24 h after SCI.

**Figure 7:**
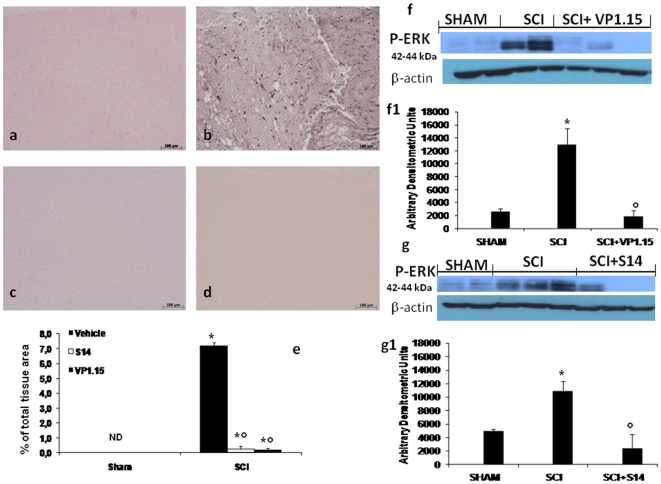
Effect of VP 1.15 and S14 on activated kinases. At 24 h after injury we assessed, by immunohistological staining, an increase of Cox-2 expression. A substantial increase in Cox-2 expression was found in spinal cord tissue collected from SCI mice group (b),whereas treatment with VP1.15 (c) and S14 (d) reduced markedly a staining for Cox-2. Densitometry analysis of immunocytochemistry photographs (n = 5 photos from each sample collected from all mice in each experimental group) for Cox-2 (e) from spinal cord tissues was assessed. The assay was carried out by using Optilab Graftek software on a Macintosh personal computer (CPU G3-266). Data are expressed as % of total tissue area. This figure is representative of at least 3 experiments performed on different experimental days. *p<0.01 vs. Sham. °*p*<0.01 vs SCI+vehicle. ND: not detectable. SCI caused an increase of the ERK1/2 phosphorylation in vehicle-treated mice (f, f1 and g, g1). The treatment with VP1.15 (f, f1) and S14 (g, g1) reduced pERK1/2 levels. Densitometric analysis of protein expression represents the mean ± s.e.mean of 10 spinal cord tissues. Data were normalized on the basis of ERK-2 levels. *P<0.01 vs sham; °P<0.05 vs SCI + vehicle.

Spinal cord sections were taken at 24 h after SCI to determine the immunohistological staining for COX-2 expression. There was no staining for COX-2 in spinal cord obtained from the sham mice (Figure 7a, see densitometry analysis Figure 7e). A substantial increase in COX-2 expression was found in inflammatory cells of the spinal cord tissues collected from SCI mice 24 hours after SCI (Figure 7b, see densitometry analysis Figure 7e) Spinal cord expression of COX-2 were significantly attenuated in mice treated with VP1.15 (Figure 7c, see densitometry analysis Figure 7e) and S14 (Figure 7d, see densitometry analysis Figure 7e) in comparison to SCI animals.

To investigate whether the increase in COX-2 expression, observed in our experimental conditions, corresponded to an activation of signal transduction pathways involved in the regulation of COX-2 expression, we analyzed the activation of MAPK pathways in particular the phosphorylation of ERK1/2 24 h after SCI by western blot analysis. As shown in Figure 6, pERK1/2 levels were significantly increased in the spinal cord tissues of SCI mice group (Figures 7f, 7f1 and 7g, 7g1). Treatment with VP 1.15 (Figures 7f, 7f1) and S14 (Figures 7g, 7g1) significantly reduced the level of pERK1/2.

### Effects of PDE7 inhibitors on iNOS formation after SCI

**Figure 8:**
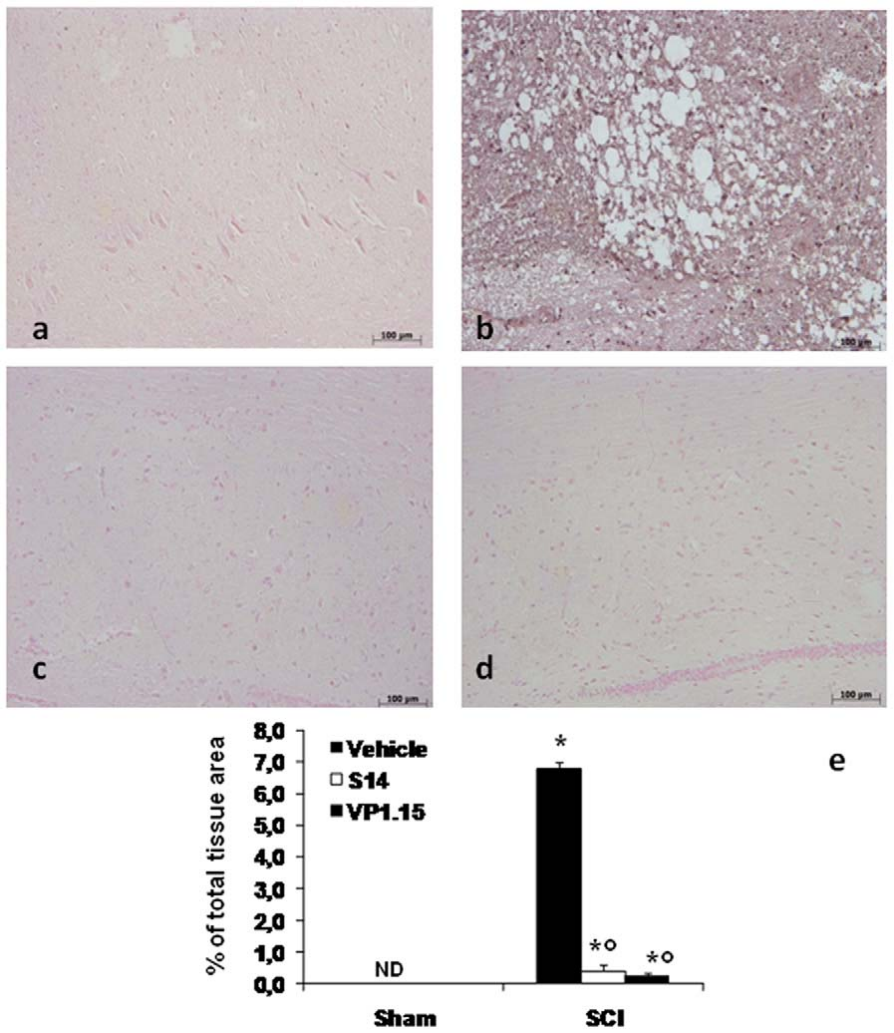
Effects of PDE7 inhibitors on iNOS expression. iNOS expression was evaluated by immunohystochemical analysis in the spinal cord section 24 h after SCI. Spinal cord section from sham-operated mice did not stain for iNOS (a), whereas spinal cord section obtained from SCI-operated mice exhibited positive staining for iNOS (b) mainly localized in various inflammatory cells in the grey matter. VP 1.15 and S14 treatment (c, d respectively) reduced the degree for positive staining for iNOS in the spinal cord tissue. Densitometry analysis of immunocytochemistry photographs (n = 5 photos from each sample collected from all mice in each experimental group) for iNOS (e) from spinal cord tissues was assessed. The assay was carried out by using Optilab Graftek software on a Macintosh personal computer (CPU G3-266). Data are expressed as % of total tissue area. ND: not detectable. This figure is representative of at least 3 experiments performed on different experimental days. *p<0.01 vs Sham. °*p*<0.01 vs SCI+vehicle.

Twenty-four hours after SCI, iNOS, a specific marker of oxidative stress, was measured by immunohistochemical analysis in the spinal cord sections. Spinal cord sections from sham-operated mice did not stain for iNOS (Figure 8a, see densitometric analysis Figure 8e), whereas spinal cord sections obtained from SCI mice exhibited positive staining for iNOS (Figure 8 b, see densitometric analysis Figure 8e). The positive staining was mainly localized in inflammatory cells in the white and gray matter of the spinal cord tissues. VP 1.15 (Figure 8c, see densitometric analysis Figure 8e) and S14 (Figure 8d, see densitometric analysis Figure 8e) treatment reduced the degree of positive staining for iNOS in the spinal cord.

### Western blot analysis and immunohistochemistry for Bax and Bcl-2

**Figure 9:**
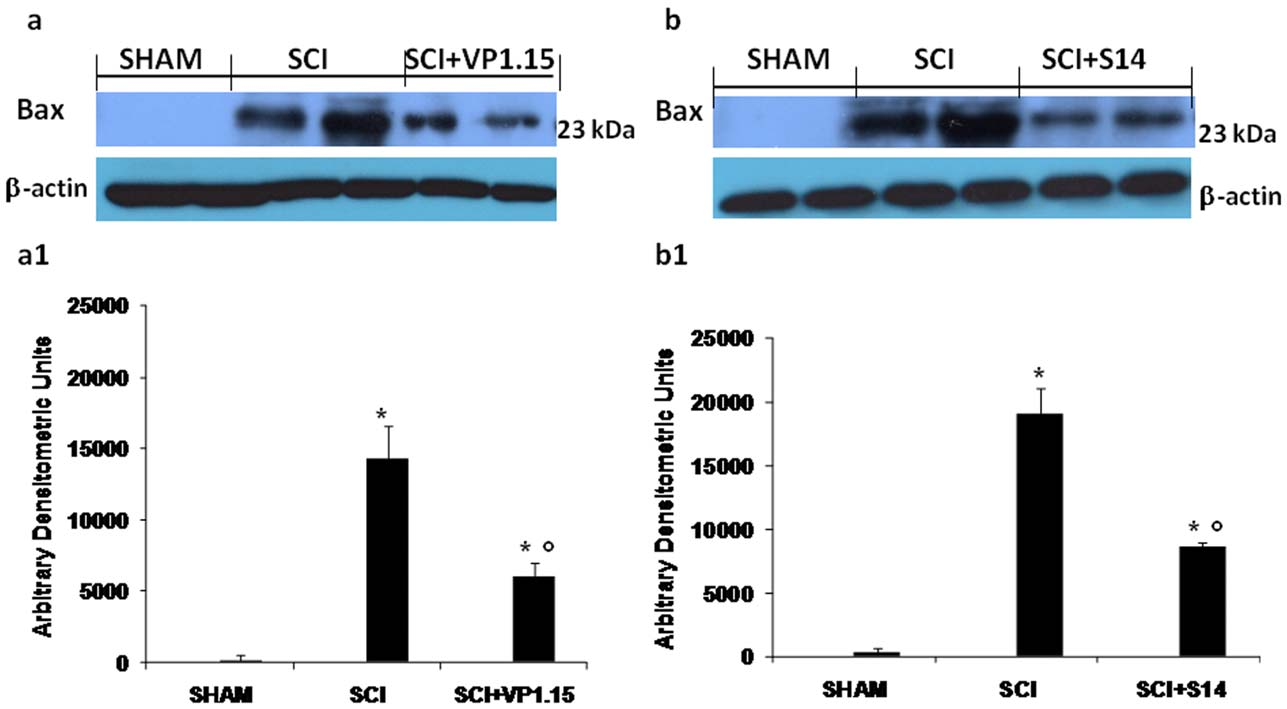
Western blot analysis for Bax. By Western blot analysis, Bax levels were appreciably increased in the spinal cord from SCI mice (a, a1 and b, b1). On the contrary, VP1.15 (a, a1) and S14 (b, b1) treatment prevented the SCI-induced Bax expression. The relative expression of the protein bands was standardized for densitometric analysis to β-actin levels, and reported in panel a, a1 and b, b1 are expressed as mean ± s.e.m. from n = 5/6 spinal cord for each group. *P<0.01 vs sham, °*P*<0.01 vs SCI+vehicle.

To test whether spinal cord damage was associated to cell death by apoptosis, we measured the levels of Bax and Bcl-2 expression. At 24 h after SCI, the appearance of pro-apoptic protein Bax, in spinal cord homogenates was investigated by Western blot. Bax levels were appreciably increased in the spinal cord from mice subjected to SCI (Figures 9a, 9a1 and 9b, 9b1). On the contrary, VP 1.15(Figures 9a, 9a1) and S14 (Figures 9b, 9b1) treatment prevented the SCI-induced Bax expression.

**Figure 10:**
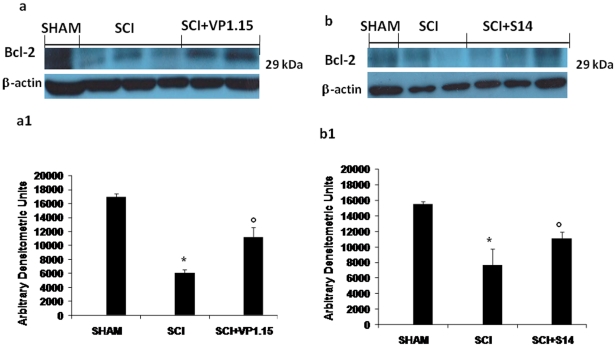
Western blot analysis for Bcl-2. Moreover, a basal level of Bcl-2 expression was detected in spinal cord from sham-operated mice (a, a1 and b, b1). Twenty-four hours after SCI, Bcl-2 expression was significantly reduced in spinal cord from SCI mice (a, a1 and b, b1). Treatment with VP 1.15 (a, a1) and S14 (b, b1) significantly reduced the SCI-induced inhibition of Bcl-2 expression. The relative expression of the protein bands was standardized for densitometric analysis to β-actin levels, and reported in panel a, a1 and b, b1 are expressed as mean ± s.e.m. from n = 5/6 spinal cord for each group. *P<0.01vs sham, °*P*<0.01 vs SCI+vehicle.

Bcl-2 expression was also analyzed by Western blot in homogenates from spinal cord of each mouse. A basal level of Bcl-2 expression was detected in spinal cord from sham-operated mice (Figures 10a, 10a1 and 10b, 10b1). Twenty-four hours after SCI, the Bcl-2 expression was significantly reduced in spinal cord from SCI mice (Figures 10a, 10a1 and 10b, 10b1). Treatment of mice with VP 1.15 (Figures 10a, 10a1) and S14 (Figures 10b, 10b1) significantly blunted the SCI-induced inhibition of anti-apoptotic protein expression.

### S14 and VP1.15 penetrate into the brain in an experimental model

**Figure 11:**
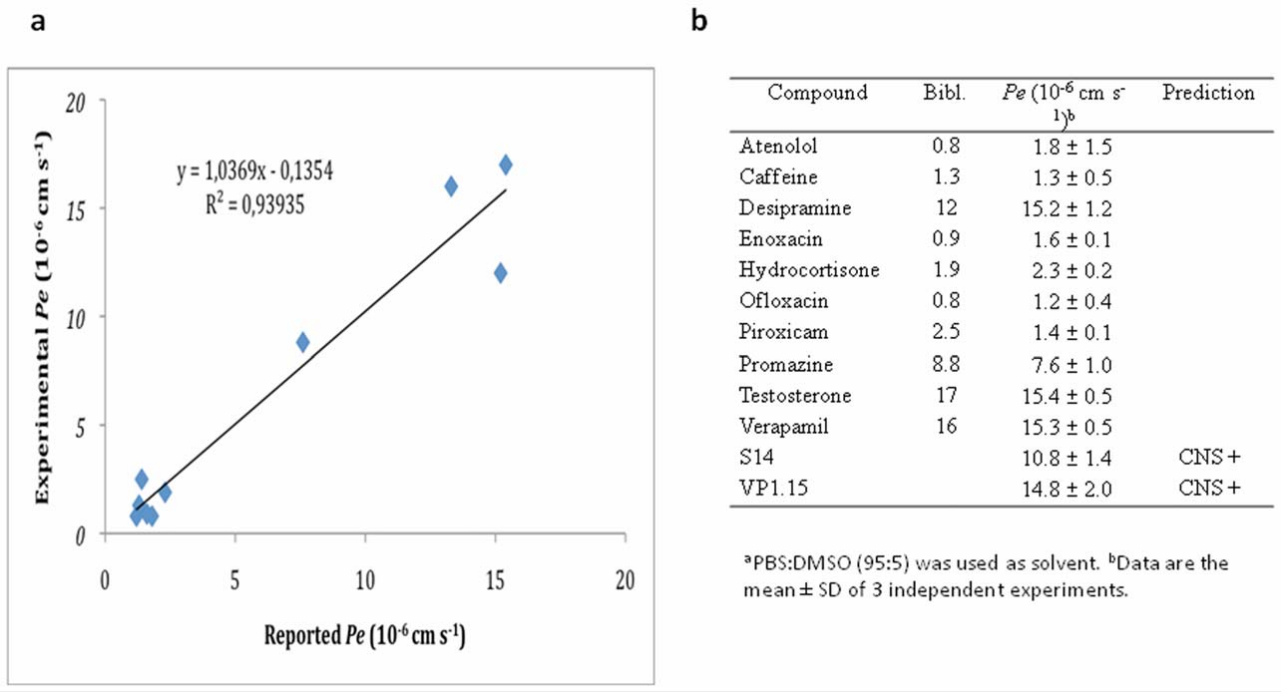
PAMPA-BBB assay results. A linear correlation between experimental and reported permeability of selected commercial drugs using the experimental conditions described in the PAMPA-BBB assay methodology was found (10a). The permeability (*Pe* 10^−6^ cm s^−1^) value obtained in the PAMPA-BBB assay for the 10 commercial drugs and used in the experiment validation, were reported in Figure 10b. The *Pe* obtained in the same experimental conditions for compounds S14 and VP1.15 predict their penetration in the CNS.

One of the main obstacles for the treatment of the diseases of the central nervous system (CNS) is the drug's penetration into the blood-brain barrier (BBB) at therapeutic concentrations. The BBB is a complex interface between blood and the central nervous system that strictly controls the exchanges between the blood and brain compartments [35]. This barrier is composed by endothelial cells with tight junctions that protect the brain from endogenous materials which could damage the brain tissues. [36] The majority of CNS drugs enter the brain by transcellular passive diffusion, due to the tight junction structure and limited transport pathways. In early drug discovery stage, evaluation of ADME (Absorption, Distribution, Metabolism, and Excretion) properties is of crucial importance to reduce attrition in development process. PAMPA assay is a high throughput technique developed to predict passive permeability through biological membranes. In order to explore the capacity of S14 and VP1.15 to penetrate into the brain, we used the PAMPA-BBB method described by Di et al [34] which employed a brain lipid porcine membrane. The *in vitro* permeabilities (*Pe*) of commercial drugs through lipid membrane extract together with compounds S145 and VP1.15 were determined and described in Figure 11b. An assay validation was made comparing the reported permeabilities values of commercial drugs with the experimental data obtained employing this methodology. A good correlation between experimental-described values was obtained (*Pe* (exptl) = 1.0369, *Pe* (bibl) = −0.1354, R^2^ = 0.939, see Figure 11a). From this equation and following the pattern established in the literature for BBB permeation prediction [37] we could classify compounds as CNS + when they present a permeability >4.01×10^−6^ cm s^−1^. Based on these results we can consider that compounds S14 and VP1.15 that are able to cross the BBB by passive permeation (Figure 11b).

## Discussion

SCI induces lifetime disability, and no suitable therapy is available to treat victims or to minimize their suffering. We report here that the pharmacological inhibition of PDE7 isoenzyme using two new chemically diverse small molecule inhibitors exerts a protective effect against the pathological changes caused by SCI. Thus, we propose that PDE7 contributes to the pathophisiology of SCI.

SCI in mice induced by the application of vascular clips (force of 24 g) to the dura via a four-level T5-T8 laminectomy, resulted in severe trauma characterized by edema, neutrophil infiltration and loss of myelin in lateral and dorsal funiculi. This histological damage was associated to the loss of motor function. SCI induced an inflammatory response in the spinal cord characterized by increased IkB-degradation, production of a range of inflammatory mediators such iNOS, and COX-2 and increased MPO activity. Treatment of the mice with our new PDE7 inhibitors named S14 and VP1.15, derivatives of quinazoline and 5-imino-1,2,4-thiadiazole scaffolds respectively, significantly reduced the degree of 1) spinal cord inflammation and tissue injury (histological score); 2) neutrophil infiltration (myeloperoxidase activity); 3) inducible nitric-oxide synthase, and cyclooxygenase-2 expression; and 4) and apoptosis (Bax and Bcl-2 expression). What is then the mechanism by which inhibition of PDE7 decrease the secondary inflammation caused by SCI? First, we have been shown previously that S14 and VP1.15 inhibit PDE7 [25], [26], one of the isoenzymes of PDEs family responsible for the degradation of cAMP and selectively expressed on macrophagues and brain [38], [39]. We have also previously shown that S14 and VP1.15 treatment of human neuroblastoma and rat macrophagues cell lines, SH-SY5Y and D10.G4.1 respectively, with these compounds lead to an increase in intracellular cAMP level [23b, 23c]. It is recently shown that elevation of endothelial cell cAMP levels inhibits degradation of IκB-α by a PKA independent mechanism[40]. In the present work we have shown a basal level of IκB-α in the spinal cord from sham-operated animals, whereas IκB-α levels were subtantially reduced in SCI mice. The prevention of SCI-induced IκB-α degradation observed in mice treated with S14 and VP 1.15 should be then attributed to the increase on cAMP.

The second possible mechanism by which S14 and VP1.15 may protect the spinal cord is as an anti-inflammatory one. We found that levels of TNF-α and IL-1β had significantly decreased in the S14 and VP1.15 treated groups. Primary injury to the adult spinal cord is irreversible, whereas secondary degeneration is delayed and therefore amenable to intervention. Accordingly, several studies have shown that therapies targeting various factors involved in the secondary degeneration cascade lead to tissue sparing and improved behavioral outcomes in spinal cord-injured animals [41], [42], [43], [44]. In this report we demonstrate that VP1.15 and S14, PDE7 inhibitors, exerts beneficial effects in a mice model of spinal cord injury. We demonstrate here that SCI resulted in edema and loss of myelin in lateral and dorsal funiculi. This histological damage was associated to the loss of motor function. SCI induced an inflammatory response in the spinal cord, characterized by increased IκB-α degradation, enhanced NF-κB activation, amplified expression of pro-inflammatory mediators, pro-inflammatory cytokines and nitrotyrosine and increased MPO activity. Our results show that VP1.15 and S14 reduced (1) the degree of spinal cord damage, (2) neutrophils infiltration, (3) IκB-α degradation, (4) nitrotyrosine formation, (5) pro-inflammatory cytokines production, and (6) apoptosis as Bax and Bcl-2 expression.

The ERK1/2 and p38 MAPK signaling pathways have been found to be involved in microglial/macrophage activation [45], [46], [47]. Previous studies show that the expression of activated ERK1/2 and p38 MAPK in microglia/macrophages may play a key role in production of CNS inflammatory cytokines and free radicals, such as NO [48], [49]. In the present study, we have observed an increase of phosphorylated MAPKs (ERK, p38, and JNK) in the spinal cord tissues at 24 h after SCI which are significantly reduced by the treatment with VP1.15 and S14. Recent evidence suggests that the activation of NF-κB may also be under the control of oxidant/antioxidant balance [50]. Moreover, various experimental evidence have clearly suggested that NF-κB plays a central role in the regulation of many genes responsible for the generation of mediators or proteins in secondary inflammation associated with SCI [51]. NF-κB is normally sequestered in the cytoplasm, bound to regulatory proteins IκBs. In response to a wide range of stimuli including oxidative stress, infection, hypoxia, extracellular signals, and inflammation, IκB is phosphorylated by the enzyme IκB kinase [52]. The net result is the release of the NF-κB dimer, which is then free to translocate into the nucleus. The exact mechanisms by which PDE7 inhibitors suppress NF-κB activation in inflammation are not known. We report here that SCI caused a significant increase in the phosphorylation of Ser536 on p65 in the spinal cord tissues at 24 h, whereas S14 and VP1.15 treatment significantly reduced this phosphorylation. Moreover, we also demonstrate that PDE7 inhibitors inhibited the IκB-α degradation as well as the NF-κB translocation. Taken together, the balance between pro-inflammatory and pro-survival roles of NF-κB may depend on the phosphorylation status of p65, and MAPK play a central role in this process. In this regard, recently it has been demonstrated that the elevation of cell cAMP levels, inhibits NF-κB activation by targeting p38 mitogen activated protein kinases (MAPK) [53]. Thus, the activity of PDE7 inhibitors on the cAMP levels might account for its effect on NF-κB activation, since have been showed that cAMP also activates protein kinase A, which inhibits NF-κB [54].

NF-κB plays a central role in the regulation of many genes responsible for the generation of mediators or proteins in inflammation. These include the genes for TNF-α, IL-1β, iNOS and COX-2, to name but a few [55]. In this regard, it has been well demonstrated that in SCI the expression of pro-inflammatory cytokines (TNF-α and IL-1β) at the site of injury regulates the precise cellular events after SCI [56], [57]. We have clearly confirmed a significant increase in TNF-α and IL-1β in SCI. On the contrary, no significant expression of TNF-α and IL-1β was observed in the spinal cord sections obtained from SCI-operated mice which received VP1.15 and S14 treatment suggesting that PDE7 pathway play an important role in the regulation of proinflammatory cytokines. This observation is in agreement with previous studies in which have been demonstrated that S14 and VP 1.15 treatment reduced the inflammatory activation of primary cell cultures of neurones, microglia and astrocytes treated with lipopolisacharide (LPS) measured by the decrease on nitrite production.

Several studies suggest that glial cells in neurodegenerative diseases (i.e., Alzheimer's disease) are affected more than neurons by apoptotic cell death [58], [59]. Apoptosis is an important mediator of secondary damage after SCI [60], [61]. It incurs its affects through at least two phases: an initial phase, in which apoptosis accompanies necrosis in the degeneration of multiple cell types and a later phase, which is predominantly confined to white matter and involves oligodendrocytes and microglia [62]. Chronologically, apoptosis initially occurs 6 hours post-injury at the lesion center and last for several days associated with the steadily increased number of apoptotic cells in this.

Various studies have postulated that preserving Bax, a pro-apoptotic gene, plays an important role in developmental cell death [63] and in CNS injury [64]. Similarly, it has been shown that the administration of Bcl-xL fusion protein (Bcl-xL FP), (Bcl-2 is the most expressed antiapoptotic molecule in adult central nervous system) into injured spinal cords significantly increased neuronal survival, suggesting that SCI-induced changes in Bcl-xL contribute considerably to neuronal death [65]. Based on these evidences, we have identified in SCI proapoptotic transcriptional changes, including upregulation of proapoptotic Bax and down regulation of antiapoptotic Bcl-2, by immunohystochemical staining. We report in the present study that the pharmacological inhibition of PDE7 pathway by VP1.15 and S14 in SCI experimental model documents features of apoptotic cell death after SCI, suggesting that protection from apoptosis may be a prerequisite for regenerative approaches to SCI. In particular, we demonstrated that the treatment with VP1.15 and S14 reduced Bax expression; while on the contrary, Bcl-2 expressed much more in mice treated with VP1.15 and S14. A lot of number of studies has linked apoptosis to SCI.

However is not possible to exclude that anti- apoptotic effect observed after VP1.15 and S14 treatment it may be partially dependent on the attenuation of the inflammatory-induced damage. Further studies are needed in order to clarify these mechanisms.

Finally, we have shown that our two new drugs VP1.15 and S14 are able to cross the blood brain barrier which increase the value of these compounds as potential candidates for further pharmacological development.

In summary, we have demonstrated that VP1.15 and S14 treatment significantly reduced the SCI-induced spinal cord tissues alteration as well as improve the motor function. The results of the present study enhance our understanding of the role of PDE7 pathway in the pathophysiology of spinal cord cell and tissue injury following trauma, implying that inhibitors of the activity of PDE7 pathway may be useful in the therapy of spinal cord injury, trauma and inflammation.
